# Determining large hyperfine interactions of a model flavoprotein in the semiquinone state using pulse EPR (electron paramagnetic resonance) techniques

**DOI:** 10.5194/mr-6-183-2025

**Published:** 2025-07-17

**Authors:** Jesús I. Martínez, Susana Frago, Milagros Medina, Inés García-Rubio

**Affiliations:** 1 Departmento de Física de la Materia Condensada, Universidad de Zaragoza, Zaragoza, 50009, Spain; 2 Departmento de Bioquímica y Biología Molecular y Celular and Instituto de Biocomputación y Física de Sistemas Complejos (BIFI), Universidad de Zaragoza, Zaragoza, 50009, Spain; 3 Instituto de Nanociencia y Materiales de Aragón, CSIC-Universidad de Zaragoza, Zaragoza, 50009, Spain; 4 Institute for Molecular Physical Science, ETH Zurich, 8093 Zurich, Switzerland

## Abstract

Flavoproteins are a versatile class of proteins involved in numerous biological processes, including redox reactions, electron transfer, and signal transduction, often relying on their ability to stabilize different oxidation states of their flavin cofactor. A critical feature of flavin cofactors is their capacity to achieve, within particular protein environments, a semiquinone state that plays a pivotal role in mediating single-electron transfer events and that is key to understanding flavoprotein reactivity.

Hyperfine interactions between the unpaired electron and magnetic nuclei in the isoalloxazine ring provide valuable insights into the semiquinone state and its mechanistic roles. This study investigates the hyperfine interactions of isotopically labeled flavodoxin (Fld) with ${}^{13}C$ and ${}^{15}N$ in specific positions of the flavin mononucleotide (FMN) ring using advanced electron paramagnetic resonance (EPR) techniques. The combination of continuous-wave (CW) EPR at the X-band and ELDOR-detected NMR and HYSCORE at the Q-band revealed a strong and anisotropic hyperfine interaction with the nucleus of ${}^{13}C$ at 4a and yielded principal tensor values of 40, -13.5 , and -9 MHz, the first of which is associated with the axis perpendicular to the flavin plane. On the other hand, as predicted, the hyperfine interaction with the ${}^{13}C$ nucleus in position 2 was minimal. Additionally, HYSCORE experiments on ${}^{15}N$ -FMN-labeled Fld provided precise axial hyperfine parameters, i.e., (74, 5.6, 5.6) MHz for ${}^{15}N$ (5) and (38, 3.2, 3.2) MHz for ${}^{15}N$ (10). These were used to refine quadrupole tensor values for ${}^{14}N$ nuclei through isotope-dependent scaling. These results showcase the potential of combining CW EPR, ELDOR-detected NMR, and HYSCORE with isotopic labeling to probe electronic and nuclear interactions in flavoproteins. The new data complete and refine the existing experimental map for the electronic structure of the flavin cofactor and expose systematic divergences between the calculated and experimental values of hyperfine couplings of the atoms that contribute most to the semi-occupied orbital (SOMO). This could indicate a slight but significant shift in the unpaired electron density from position 4a towards the central nitrogens of the pyrazine ring as compared with the calculations. These results highlight the importance of integrating computational and experimental approaches to refine our understanding of flavin cofactor reactivity.

## 1 Introduction

Flavoproteins constitute an extensive and versatile family of proteins which are involved in a wide range of biochemical and physiological processes. These mainly include oxido-reduction processes for the catalysis of different redox reactions (Minjárez-Sáenz et al., 2022; Walsh and Wencewicz, 2013) and the support of electron transfer chains and biosynthetic processes (Curtabbi et al., 2024; Minjárez-Sáenz et al., 2025; Buey et al., 2021). Moreover, some flavoproteins have the specific ability to act as sensors of the redox state of a cell, oxygen, or voltage (Fraaije and Mattevi, 2000; Villanueva et al., 2019; Tsukuno et al., 2018) and to activate catalytic processes by blue light or magnetoreception (Calloni and Vabulas, 2023; Kaya et al., 2025).

**Figure 1:**
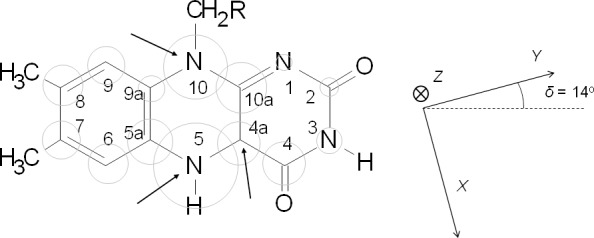
Sketch of the molecular structure and IUPAC numbering of a (7,8-dimethyl) isoalloxazine ring. Grey circles are proportional to the isotropic hyperfine interaction of ${}^{14}N$ and ${}^{13}C$ nuclei in the neutral semiquinone state. Arrows in the sketch indicate positions where experimental hyperfine interactions are determined in this work. The principal axes of the g tensor are displayed on the right. The orientation of the g tensor was taken from Kay et al. (2005) and used in the simulations shown later.

Flavoproteins have for their cofactor a group that includes the 7,8-dimethyl-isoalloxazine ring, usually in the form of a flavin mononucleotide (FMN) or a flavin adenine dinucleotide (FAD) (see Fig. 1). This ring is able to reach three different oxidation states: fully oxidized, fully reduced by two electrons, and an intermediate semiquinone state holding an additional electron over the oxidized state and, therefore, having an unpaired electron and being paramagnetic. The semiquinone state is hardly detectable in redox processes involving free flavins (see Rostas et al., 2018, and references therein), but in some flavoproteins it is highly stabilized by the protein environment (hydrogen bonding and electrostatic interactions). This is particularly the case with electron-transferring flavoproteins like flavodoxin (Fld), flavoproteins containing Fld-like domains (like cytochrome P450 reductases and nitric oxide synthase), or bifurcating electron-transferring flavoproteins, which play crucial roles in anaerobic metabolism, energy conservation, and the maintenance of redox balance by efficiently distributing electrons (Mayhew and Ludwig, 1975; Iyanagi, 2005; Bestsova et al., 2019; Mohamed-Raseek and Miller, 2022; González-Viegas, 2023). This is an indication that the unique properties of the isoalloxazine ring are modulated by the protein environment in order to select its redox and electronic properties to develop different functions in each specific flavoprotein. In particular, the ability of some flavoproteins to stabilize the semiquinone state during their redox cycle makes them unique molecules for mediating processes involving donor or acceptors with the ability to exchange only one electron at a time (in general, metal centers) with those that necessarily exchange two electrons (e.g., pyridine nucleotides) (Lans et al., 2012; Young et al., 2020; Moreno et al., 2024).

The spin density distribution in the semiquinone state of flavin cofactors has a clear relation to protein reactivity, and its characterization can provide insights into electron transfer pathways. Better knowledge of the orbital composition of the semi-occupied orbital (SOMO) of the semiquinone state is of great mechanistic relevance, because the reactivity of the fully reduced state to generate it and the topology of the electron transfer process depend entirely on it. Also, the lifetime of this semiquinone intermediate and the way it transits to the completely oxidized state for processes occurring in the different flavoproteins are intimately related to such an electronic structure. Hyperfine interactions between the unpaired electron and the magnetic nuclei in the cofactor are directly related to the orbital composition of the SOMO and can be used to probe electron density. Moreover, hyperfine interactions are relevant for the elucidation of the flavoprotein mechanisms themselves as they influence, for instance, flavin magnetochemistry in magnetoreception or the lifetimes of semiquinone intermediates. Therefore, numerous reports have aimed to characterize hyperfine structures using different techniques of electron paramagnetic resonance (EPR) on several flavoproteins (Weber et al., 2005; Schleicher et al., 2009, 2021; Martínez et al., 2012; Schleicher and Weber, 2012; Brosi et al., 2014; Nohr et al., 2019).

The effect of hyperfine interactions can already be observed in the CW-EPR spectra of the flavin. However, characterizations by hyperfine spectroscopy methods able to provide higher resolution (ESEEM-HYSCORE or ENDOR) have revealed more details about the anisotropy of the interaction. The use of different techniques and microwave frequencies, together with model flavoproteins containing isotopically enriched flavins in selected positions or flavin analogs, has allowed us to obtain complete hyperfine tensors for many nuclei at the isoalloxazine ring. Most reported hyperfine interactions refer to protons bound to the ring. Although they provide relevant information, these interactions are just indirectly related to spin densities at the sites of the ring to which they are attached, and, therefore, they are less significant compared to the interactions with nuclei that are directly part of the ring. To probe the electronic distribution of the carbon atoms on the isoalloxazine, the use of flavin samples with flavins that are isotopically labeled is necessary, since the most abundant carbon nucleus ${}^{12}C$ has no nuclear spin; i.e., $I_{\text{12C}}=0$ . For nitrogen positions, over 99 % of nuclei are ${}^{14}N$ , with a nuclear spin of $I_{\text{14N}}=1$ that usually shows experimental evidence that is difficult to interpret because of the presence of quadrupole interactions and many nuclear transitions and correlations (see the “Materials and methods” and “Spin Hamiltonian” sections below). Therefore, labeling with ${}^{15}N$ ( $I_{\text{15N}}=1/2$ ) is also helpful. As a result of the combination of high-resolution methods and isotopic labeling, some information on couplings to all of the nitrogen nuclei in the ring has been obtained through HYSCORE experiments at the X-band, CW EPR at the W-band, and pulse ENDOR at the W-band (Martínez et al., 1997; Barquera et al., 2003; Weber et al., 2005). Additionally, a detailed study has been published on a protein with a flavin cofactor selectively enriched with ${}^{13}C$ at different carbon sites, using the pulse ENDOR technique at the W-band (Schleicher et al., 2021). However, the characterization of the hyperfine interaction with some of the nuclei at the sites with the highest spin densities, i.e., positions C(4a) and N(5) (see Fig. 1 for the site numbering), still remains incomplete, preventing the experimental determination of the spin population. This information is also of particular interest from the flavoprotein and flavoenzyme functional points of view, because the N(5)–C(4a) locus of the flavin concentrates most of its chemical prowess (Lans et al., 2012; Beaupre and Moran, 2020). In fact, the N(5) of the isoalloxazine is known to be relevant during redox processes and substrate oxygenation, while C(4a) is a recognized site for one-electron chemistry during flavin reoxidation processes (Beaupre and Moran, 2020; Sucharitakul et al., 2011; Ghisla and Massey, 1989; Visitsatthawong et al., 2015; Saleem-Batcha et al., 2018). Furthermore, the reactivity of N(5) and C(4a) allows formation of covalent intermediates contributing to increases in the chemical repertoire of natural flavin derivatives within flavoproteins (Leys and Scrutton, 2016; Beaupre and Moran, 2020).

In parallel to these studies, calculations, mainly based on density functional theory (DFT) computational methods, have been published in the last 25 years to improve the knowledge of the electronic structure of the flavin cofactor in its three oxidation states (HOMO and LUMO of the completely oxidized and reduced states and SOMO of the semiquinone state), aiming to predict their physicochemical properties (Domratcheva et al., 2014). Although these methods have a great capacity to develop realistic electronic structures, considering in detail the effect of the environment, obviously their validity must be contrasted with experimental results and the discrepancy must be used to improve or refine the results of those calculations. Hyperfine interactions of the unpaired electron in the semiquinone state with nuclei in the vicinity of the isoalloxazine ring are very valuable experimental control parameters for contrasting the results of the calculations, as these interactions are directly related to the spin density distribution in the SOMO. They provide an appropriate tool for testing the predictions of the calculations for the electronic structure of the semiquinone state, as the magnetic nuclei act as local probes for drawing a map of the electronic spin density within the ring.

Over these years it has been recognized through the calculation of different flavoproteins (such as Fld and DNA photolyase) that the values for the hyperfine parameters obtained are very sensitive to the level of calculation, the functional used, and the environment of the flavin that is considered (Weber et al., 2001; García et al., 2002; Schleicher et al., 2021). However, experimental evidence indicates that hyperfine interactions are very similar in different flavoproteins (Barquera et al., 2003; Martínez et al., 2012; Schleicher and Weber, 2012; Paulus et al., 2014; Nohr et al., 2019; Pompe et al., 2022) and that the substitution of residues in the flavin environment or in the ring atoms barely affects the hyperfine splittings (Medina et al., 1999; Martínez et al., 2016). In general, the experimental differences between different flavoproteins are on the same order or smaller than those that appear between calculations, depending on their technical details (level, functional used, and environment considered). The agreement between the calculations and the known experimental values of $a_{\text{iso}}$ is fair for interactions with ${}^{1}H$ nuclei (most of the calculated values are within ±20 % of the experimental values), but the hyperfine couplings with ${}^{14}N$ (5) and ${}^{14}N$ (10) nuclei appear to be systematically underestimated, whereas the relative error remains within ±20 % for the completely characterized ${}^{13}C$ nuclei within the flavin ringThe relative error for some weakly coupled nuclei is larger than this value. However, since the absolute values of the discrepancies are small, they are not considered to be very relevant. (Schleicher et al., 2021).

In the analysis of the hyperfine interactions describing the electronic structure of the flavin semiquinone radical for a better understanding of its role in flavoprotein-catalyzed reactions, it is certainly worth including the hyperfine interactions with nitrogen nuclei. Nitrogen atoms occupy four positions within the ring, i.e., N(1), N(3), N(5), and N(10), for which previously reported experimental evidence of the hyperfine couplings is available (Martínez et al., 1997, 2012; Barquera et al., 2003; Weber et al., 2005). Furthermore, the anisotropic part of the hyperfine couplings provides relevant information that should not be neglected. In a pure π radical, the hyperfine interaction with nuclei on the ring is axial; i.e., the axis is perpendicular to the plane of the ring. The detection of orthorhombic hyperfine matrices or hyperfine principal values that show different proportions between the isotropic and anisotropic parts implies a mixture of π and σ orbitals linked to distortion of the molecular and/or electronic structure that can have a relevant effect on the mechanisms where flavin is involved, as has been shown in Fld variants where the naturally occurring FMN cofactor has been substituted with different analogs (Lans et al., 2012; Martínez et al., 2016). In addition, although this evidence does not directly inform the electronic structure of the fully reduced and oxidized states, the disparity between the calculated and measured hyperfine splitting values offers an indirect indication of differences that may exist between the electronic structures calculated for those states and the real ones.

In this work we present an X-band and Q-band study of the neutral semiquinone of *Anabaena* Fld combining CW EPR, ELDOR-detected NMR, and HYSCORE experiments with selective ${}^{13}C$ and ${}^{15}N$ isotope labeling of the flavin. Fld was chosen as a model system due to its feasibility in replacing its FMN cofactor with modified flavins and its ability to stabilize a large proportion of its neutral semiquinone state (Martínez et al., 2012, 2014, 2016; Lans et al., 2012). This particular combination of experiments and microwave frequencies (about 9 and 34 GHz) has turned out to be especially suited to the detection of couplings in the C(4a), N(5), and N(10) positions of the isoalloxazine, allowing complete characterization of the hyperfine interactions for the nuclei ${}^{13}C$ (4a), ${}^{15}N$ (5), and ${}^{15}N$ (10). These results provide a suitable protocol for experimentally accessing these couplings and thus estimating the spin density distribution in the isoalloxazine ring for the Fld model system.

The results are discussed based on the predictions of published calculations and our knowledge of electron transfer processes involving flavoproteins.

## 2 Materials and methods

### 2.1 Biological material

Riboflavin (RF) analogs ${}^{13}C$ (2)-RF and ${}^{13}C$ (2,4a)-RF were converted into the corresponding FMN forms using the mutant H28A of FAD synthase (FADS) from *Corynebacterium ammoniagenes* (Frago et al., 2008, 2010). Reaction mixtures containing 50 µM of the RF analog, 0.5 mM ATP, 1 mM ${\mathrm{MgCl}}_{2}$ , and 1.5–3 µM H28A FADS in 50 mM Tris / HCl at pH 8.0 were incubated in the dark at 37 °C overnight. Full conversion of RF into FMN was checked by thin-layer chromatography in silica-gel plates. Once the reaction was completed, FADS was separated from the flavin by ultrafiltration (Amicon Ultra, Millipore, 10 000 MW cutoff). ${}^{15}N$ -labeled FMN was produced as previously described (Martinez et al., 1997). *Anabaena* Fld (ApoFld) was over-expressed in *Escherichia coli* and purified as described in Genzor et al. (1996). ApoFld was prepared by treatment with 3 % trichloroacetic acid at 4 °C in the presence of dithiothreitol. The precipitated apoprotein was separated from FMN by centrifugation and dissolved in 500 mM MOPS pH 7.0 before dialysis against 50 mM MOPS pH 7.0. Finally, *Anabaena* ApoFld was incubated with a 1.5-fold molar excess of each FMN analog (i.e., ${}^{13}C$ (2)-FMN, ${}^{13}C$ (2,4a)-FMN, or ${}^{15}N$ -FMN) in 50 mM MOPS at pH 7.0 for 1 h at 25 °C . Excess flavin was then removed by ultrafiltration and the reconstituted Flds stored at -20 °C . Samples with a protein concentration of 400–800 µM in 50 mM MOPS at pH 7.0 were placed in 3 mm EPR tubes and anaerobically reduced in an argon atmosphere to the semiquinone state at 4 °C by light irradiation with a 150 W barr and stroud light source approximately 7.5 cm from the sample in the presence of 20 mM EDTA and 2.5 µM 5-deazariboflavin. Once maximal production of the neutral semiquinone was obtained, samples were frozen and stored in liquid nitrogen (at 77 K) until use in EPR measurements.

### 2.2 EPR spectroscopy

X-band EPR experiments were performed using a Bruker ELEXSYS E580 spectrometer (microwave (mw) frequency ∼9.7 GHz) equipped with a cylindrical dielectric cavity and a helium gas-flow cryostat from Oxford Inc. Q-band pulse EPR measurements were carried out using a home-built spectrometer operational in the frequency range of 34.5–35.5 GHz (Gromov et al., 2001) and a Bruker ELEXSYS E580 able to work at the X-band and Q-band. Both spectrometers were equipped with custom-made resonators allowing the use of 3 mm sample tubes (Tschaggelar et al., 2009). The spectra were taken at 50 or 90 K. The repetition rate was generally 3 kHz. HYSCORE and ELDOR-detected NMR experiments were carried out in different observer positions that correspond to different selections of orientations of the molecules with respect to the magnetic field.

#### 2.2.1 CW-EPR and other field-swept spectra

The X-band CW-EPR spectra were acquired at a mw frequency of 9.714 GHz and a temperature of 50 K, using a modulation amplitude of 2 G and a mw power of 0.32 µW . The Q-band electron spin echo (ESE)-detected EPR spectra were detected using a π/2 – τ – π – τ –*echo* sequence with pulse lengths of 16 and 32 ns for the π/2 and π pulses. The Q-band free induction decay (FID)-detected EPR spectra were recorded with the pulse sequence π –FID, where the mw pulse was 1 µs and the FID was integrated for its entire duration.

#### 2.2.2 HYSCORE

HYSCORE experiments (Höfer, 1994; Schweiger and Jeschke, 2001) were performed at Q-band frequency (34.3 GHz) at temperatures of 50 K ( ${}^{13}C$ -labeled samples) or 90 K ( ${}^{15}N$ -labeled samples) using the pulse sequence π/2 – τ – π/2 – $t_{1}$ – π – $t_{2}$ – π/2 – τ –*echo*. Different τ values were used and specified in the corresponding figure captions. Unless stated otherwise, a pulse length of 12 ns for all of the pulses was used in the experiments performed at the echo maximum to obtain a maximum excitation width, and 24 and 16 ns pulse lengths were programmed for the π/2 and π pulses, respectively, for the experiments performed at the high-field or low-field flanks of the CW spectrum in order to obtain better orientation selectivity. The time intervals $t_{1}$ and $t_{2}$ were varied in steps of 8, 12, or 16 ns starting from 96 ns. A standard phase cycle of eight steps (Gemperle et al., 1990) was used to eliminate unwanted echoes. The experimental time traces were baseline-corrected, apodized with a Hamming or Gaussian window, and zero-filled. After a Fourier transformation in the two time dimensions, the absolute-value spectra were calculated and plotted with MATLAB.

#### 2.2.3 ELDOR-detected NMR

The Q-band ELDOR-detected NMR (Schosseler et al., 1994; Schweiger and Jeschke, 2001) experiments were performed using the pulse sequence (HTA)_mw2_– τ –( π )_mw1_–FID. Two rectangular pulses were used. The pulse lengths were 1 µs for the first pulse with a variable mw frequency (mw_2_) and 1 µs for the second pulse with a fixed mw frequency (mw_1_). The length and power of the detection pulse were set by optimizing the FID-integrated intensity, whereas the ELDOR pulse length and power (which were finally set using an ELDOR channel attenuation of 0 dB) were chosen by optimizing the ELDOR-detected NMR spectra. The separation between the end of the high turning angle (HTA) pulse and the beginning of the π pulse was τ=1.5 µs . The FID generated after the second pulse was integrated over a width of 800 ns. The real and imaginary parts were acquired and baseline-shifted, and the absolute value was calculated. The spectra were inverted (multiplied by -1 ) for display.

### 2.3 Spectral simulations

HYSCORE spectra were simulated using the toolbox for MATLAB EasySpin (Stoll and Schweiger, 2006) version 6.0.6, which is freely downloadable from https://www.easyspin.org (last access: 9 July 2025), and using the functions *pepper* and *saffron* together with the spin Hamiltonian specified below. For the HYSCORE simulations, in a first step, the nuclear frequencies of individual nuclei were computed using the *endorfreq* function and the Hamiltonian given in Eq. (1), whereby the orientation selection of the experiment was taken into account. From these frequencies, the position and shape of the HYSCORE correlation ridges of the individual nuclei can be deduced, but no information is obtained about the intensity of the cross-peaks. In a second step, once all of the transitions were identified, HYSCORE simulations of either a single nucleus or a set of nuclei were performed using the *saffron* function of EasySpin, which provides the intensities of all the ridges, including the combination lines that may appear when two or more nuclei are simulated. The time-domain simulations were processed and plotted using the same procedure as for the experimental data described above.

### 2.4 Spin Hamiltonian

The spin Hamiltonian (SH) that was used to analyze the experimental spectra and characterize the hyperfine interactions of the semiquinone radical ( S=1/2 ) with n different nuclear spins ( $I_{j}$ ) in the isoalloxazine ring consists of several terms.

1 $$\begin{array}{rl} H= & \mu_{B}B\hat{g}S+\sum_{j}\mu_{N}Bg_{Nj}I_{j}+\sum_{j}S{\hat{A}}_{j}I_{j}\\ & +\sum_{I_{j}>1/2}I_{j}{\hat{Q}}_{j}I_{j} \end{array}$$

The first and second terms of the SH represent the electron and nuclear Zeeman interactions, respectively. In the case of semiquinone radicals, the electron $\hat{g}$ tensor is close to the free-electron g factor, as expected for a radical. However, at higher mw frequencies, some anisotropy in the tensor can be resolved (Fuchs et al., 2002; Kay et al., 2005; Okafuji et al., 2008). In this article we assumed ( $g_{Z}=2.0022$ , $g_{Y}=2.0036$ , and $g_{X}=2.0043$ ), and the principal axes are as shown in Fig. 1 (Kay et al., 2005). The third term takes into account the hyperfine interactions with the different magnetic nuclei. If I>1/2 , as is the case for ${}^{14}N$ ( I=1 ), the nuclear–quadrupole interaction has to be included (fourth term). ${\hat{A}}_{j}$ and ${\hat{Q}}_{j}$ are the hyperfine and nuclear quadrupole tensors, respectively, of nucleus j .

**Figure 2:**
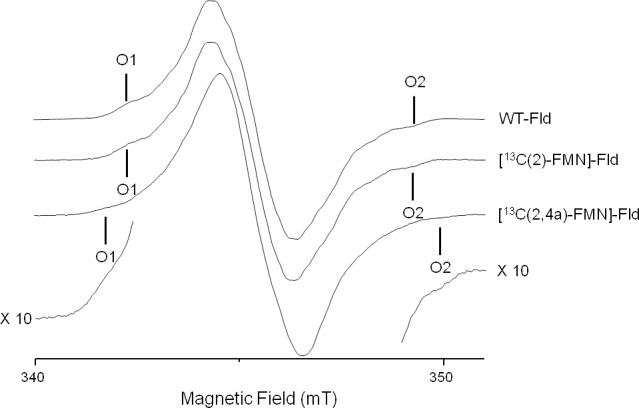
X-band CW-EPR spectra of ${}^{13}C$ -labeled Fld variants at 50 K.

Reflecting the planar symmetry of the molecule, the hyperfine tensors of magnetic nuclei directly on the flavin ring have been reported to be mostly axial, with the distinct axis perpendicular to the ring (normally called z ). This z axis coincides with the Z principal axis of the $\hat{g}$ tensor. Since most of the electron density is located in π orbitals, the hyperfine interaction along this direction is usually noticeably larger than in the other two directions contained in the plane. For moderate mw frequencies (X-band and below), this results in a CW-EPR spectrum with a central intense line corresponding to the perpendicular features and low-field and high-field wings that are contributed mainly by the molecules oriented with their axes parallel to the magnetic field. On these wings, some ripples due to large (parallel) hyperfine couplings can be resolved (Martínez et al., 2016). The distance between the two outermost features (the highest field, labeled O2 in Fig. 2, and the lowest field, labeled O1 in Fig. 2) is the sum of the couplings of all magnetic nuclei in the direction perpendicular to the isoalloxazine plane. For flavins with natural isotopic abundance, the three nuclei with the largest couplings in semiquinone radicals are ${}^{14}N$ (5), ${}^{1}H$ (5), and ${}^{14}N$ (10). Therefore, neglecting smaller couplings, the distance between the two outermost shoulders is approximately

2 $$\begin{array}{rl} & \Delta B_{\text{out}}^{\text{wt}}=[B(\text{O2})-B(\text{O1})]\\ & \;\approx C\{2[A_{z}({}^{14}N(5))+A_{z}({}^{14}N(10))]+A_{z}({}^{1}H(5))\}, \end{array}$$

where $C=\frac{h}{g_{e}\mu_{B}}=3.57\times 10^{-2}$ $\mathrm{mT}\;{\mathrm{MHz}}^{-1}$ is a constant for translating the couplings observed in the spectrum (from mT into MHz), considering that the g factor is close to the one of the free electron, i.e., $g\approx g_{e}$ . Also, since hyperfine interactions for N(5) and N(10) are almost axial, we use $A_{\|}\equiv A_{z}$ , $A_{⊥}\equiv A_{x},A_{y}$ . Expression (2), as will be shown in the next section, can be used to estimate unknown large hyperfine couplings if the others are known or if a reference spectrum without the particular nucleus of interest is available.

## 3 Experimental results

### 3.1 Flavodoxin selectively labeled with ${}^{13}C$ in positions 2 and 4a of the FMN ring

#### 3.1.1 CW EPR

The X-band CW-EPR spectra for the [ ${}^{13}C$ (2)-FMN]-Fld and [ ${}^{13}C$ (2,4a)-FMN]-Fld samples are shown in Fig. 2. The experimental trace of non-isotopically labeled Fld (wild-type – WT – Fld), described in a previous work (Martinez et al., 2016), is also shown here for comparison. According to what was said above, if the hyperfine interaction with the ${}^{13}C$ nuclei in the labeled samples is large in the direction perpendicular to the isoalloxazine plane, the distance between the outermost O1 and O2 shoulders is predicted to increase. It can be seen that, for [ ${}^{13}C$ (2)-FMN]-Fld, the spectrum does not change with respect to the WT or the O1–O2 distance, which indicates that the coupling of the ${}^{13}C$ nucleus in position 2 is small. On the other hand, an increase in the separation between the two outermost shoulders is clearly seen for [ ${}^{13}C$ (2,4a)-FMN] Fld. This indicates that the X-band CW-EPR experiments can provide a first estimate of the hyperfine splitting due to the ${}^{13}C$ (4a) nucleus in the direction perpendicular to the isoalloxazine ring, since the broadening can be directly attributed to the hyperfine coupling of ${}^{13}C$ (4a):

3 $$\begin{array}{rl} \Delta B_{\text{out}}^{\text{13C(4a)FMN}}\approx C\{ & 2[A_{z}({}^{14}N(5))+A_{z}({}^{14}N(10))]\\ & +A_{z}({}^{1}H(5))+A_{z}({}^{13}C(\text{4a}))\}. \end{array}$$

The difference between the B (O2)– B (O1) splitting of the WT Fld (or [ ${}^{13}C$ (2)-FMN]-Fld) and [ ${}^{13}C$ (2,4a)-FMN]-Fld samples is

4 $$\begin{array}{rl} \Delta B_{\text{out}}^{\text{13C(4a)FMN}} & -\Delta B_{\text{out}}^{\text{wt}}\\ & =(8.4\pm 0.3\;\mathrm{mT})-(7.1\pm 0.3\;\mathrm{mT}),\\ & =1.3\pm 0.4\;\mathrm{mT}\approx CA_{z}({}^{13}C\text{4a}), \end{array}$$

which gives a first estimate of the hyperfine coupling in the direction perpendicular to the isoalloxazine plane $A_{z}({}^{13}C(\text{4a}))\approx 36$ MHz.

It should be noted that calculations of the hyperfine coupling of this nucleus in flavoproteins (Weber et al., 2001; García et al., 2002) indicated that it is an anisotropic and nearly axial interaction, with the largest splitting in the direction perpendicular to the plane. On the other hand, these calculations predicted a value for $A_{z}$ ( ${}^{13}C$ (4a)) that was around double that obtained in our experiment ( $A_{z}^{\text{calc}}({}^{13}C(\text{4a}))\approx 70$ –90 MHz). This discrepancy will be discussed later.

In order to gain accuracy and further information about ${}^{13}C$ (4a) hyperfine coupling, advanced EPR techniques were also used.

#### 3.1.2 ELDOR-detected NMR

The ELDOR-detected NMR technique can be used to detect nuclear frequencies in systems for which EPR transitions are partially allowed due to, for example, hyperfine anisotropy and/or quadrupole interaction. Pumping an EPR-forbidden transition with a variable frequency pulse burns a hole in the polarization that is detected by a decrement in the integrated FID intensity generated by a detection pulse when it hits an allowed EPR transition. Negative peaks associated with nuclear frequencies are obtained when plotting the echo intensity as a function of the pump frequency (ELDOR frequency) in symmetric positions with respect to the detection frequency. Since this experiment is based on driving the polarization, the signal obtained is free of blind spots and other distortions, although it has zero intensity for the principal directions of the hyperfine tensor, since in those directions EPR transitions are completely allowed.

**Figure 3:**
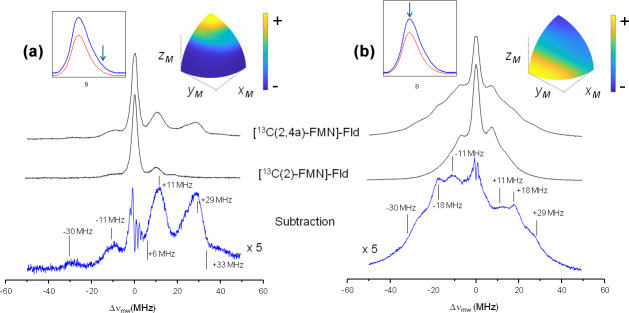
ELDOR-detected NMR spectra of ${}^{13}C$ -labeled Fld variants. **(a)** Spectra taken at the high-field tail of the Q-band echo-detected EPR spectrum, B=1225 mT, corresponding to selective excitation of molecules with the magnetic field oriented perpendicular to the isoalloxazine ring ( $A_{\|}$ since the magnetic field is parallel to the axis). **(b)** Spectra taken at the maximum of the Q-band echo-detected EPR spectra B=1221.4 mT for [ ${}^{13}C$ (2,4a)-FMN] Fld and B=1222.2 mT for [ ${}^{13}C$ (2)-FMN]-Fld, which yield poor orientation selection for all of the spectra at T=50 K. The upper insets on the left show the echo-detected EPR spectra of the samples (blue: measured; red: simulated), with the magnetic field setting of the experiment indicated by an arrow. The insets on the right show the pattern of excited orientations in a sphere octave according to the colors of the accompanying scale where the + and - signs indicate more and less populated orientations, respectively. The darkest blue actually corresponds to no contribution of those orientations to the spectrum. For the calculation of these patterns, a spin Hamiltonian containing the Zeeman anisotropic term and anisotropic hyperfine couplings of ${}^{14}N$ (5), ${}^{14}N$ (10), and ${}^{1}H$ (5) was used. See Table 1 and Fuchs et al. (2002) for the hyperfine values. The pulse excitation bandwidth corresponds to 1 MHz.

Figure 3 shows the ELDOR-detected NMR experiments of [ ${}^{13}C$ (2)-FMN]-Fld and [ ${}^{13}C$ (2,4a)-FMN]-Fld. For the ${}^{13}C$ (4a) nucleus, the values of the hyperfine coupling and the Larmor frequency at the Q-band (13.0 MHz) are comparable. Therefore, at intermediate orientations there will be partially forbidden transitions involving ${}^{13}C$ (4a) nuclear levels suitable for detection with Q-band ELDOR-detected NMR. The experiments were performed with the magnetic field set to the Q-band echo-detected EPR maximum (Fig. 3b) as well as at the high-field tail of the spectrum (Fig. 3a). As mentioned before, in the second case orientation selection occurs since only molecules oriented with the isoalloxazine plane approximately perpendicular to the direction of the magnetic field will contribute to the spectrum (Martínez et al., 2014). On the other hand, experiments with the magnetic field at the center of the spectrum are contributed by all possible orientations in the disordered sample, with a preference for molecules with the magnetic field oriented close to the isoalloxazine plane (see the insets in Fig. 3). The spectra obtained by the subtraction of the two ${}^{13}C$ labeled samples measured under identical conditions are also shown for both magnetic field positions, and the obtained lines correspond to the nuclear frequencies of the ${}^{13}C$ (4a) nucleus (Fig. 3, bottom spectra).

For the tail spectra, two wide signals are obtained, centered around ±30 and ±11 MHz. The value of the largest one is larger than $2\nu_{L}({}^{13}C)=26.2$ MHz, which indicates that the nucleus is in the strong coupling regime at this particular orientation ( $\left|A_{z}|>2\nu_{L}({}^{13}C\right)$ ). In this regime, nuclear frequencies for a nucleus with I=1/2 are approximately

5 $$\nu_{+}=\left|\frac{A_{z}}{2}+\nu_{L}\right|\;,\;\nu_{-}=\left|\frac{A_{z}}{2}-\nu_{L}\right|,$$

and therefore

6 $$\nu_{+}-\nu_{-}=2\nu_{L}\;,\;\nu_{+}+\nu_{-}=|A_{z}|.$$

Since the selection by field is not perfect (see the insets in Fig. 3), the peaks in the spectrum are contributed by molecules for which the direction perpendicular to the isoalloxazine plane presents a wide distribution of orientations around that of the magnetic field. It should also be taken into account that the direction perpendicular to the isoalloxazine plane ( z ) is likely one of the principal axes of the hyperfine tensor of this nucleus (García et al., 2002; Weber et al., 2001). Thus, molecules perfectly aligned with the field do not contribute to the ELDOR-detected NMR spectra, as they display completely allowed hyperfine transitions. Because of this, the edges of the detected broad signals (approximately 6 and 33 MHz) could then be used in Eq. (6) to provide a first estimation of the values for the nuclear transitions. The difference between these values (Eq. 5) is not far from $2\nu_{L}({}^{13}C)$ , which confirms the assignment of the peaks to the nuclear frequencies of ${}^{13}C$ (4a). Using Eq. (6), we estimate $|A_{z}[{}^{13}C(\text{4a})]|\approx 39$ MHz, which is compatible with the estimations from the X-band CW-EPR experiments. The signals being wide indicates that the hyperfine tensor of ${}^{13}C$ (4a) is quite anisotropic.

In the experiment at the center field, very wide signals are also distinguished. The main distinct feature of the spectrum is a peak at ±18 MHz. It could correspond to the perpendicular feature of the largest nuclear frequency since many of the orientations are close to the perpendicular plane. In such a case, its absolute value would be approximately $|A_{x,y}[{}^{13}C(\text{4a})]|\approx 10$ MHz. Assuming that the hyperfine tensor is very anisotropic, it is likely that $A_{z}$ [ ${}^{13}C$ (4a)] and $A_{x,y}$ [ ${}^{13}C$ (4a)] will exhibit opposite signs. The evidence from the ELDOR-detected NMR experiments does not allow resolution of the $A_{x}$ and $A_{y}$ of ${}^{13}C$ (4a), but since the hyperfine interactions in the flavin ring tend to be nearly axial, we can assume that $A_{x}$ [ ${}^{13}C$ (4a)] would be close to $A_{y}$ [ ${}^{13}C$ (4a)]. Then, a first estimation from the analysis of these experiments would be

$$A_{z}[{}^{13}C(\text{4a})]\approx +39\;\mathrm{MHz},\;A_{⊥}[{}^{13}C(\text{4a})]\approx -10\;\mathrm{MHz}.$$

These values can be refined further from evidence obtained from Q-band HYSCORE experiments.

#### 3.1.3 HYSCORE

**Figure 4:**
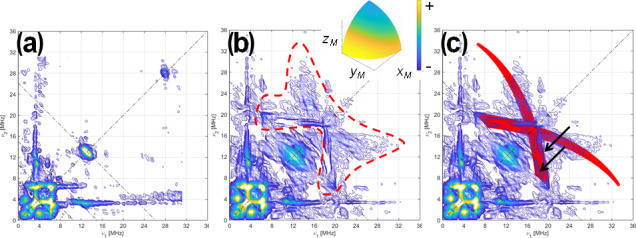
HYSCORE of ${}^{13}C$ -labeled Fld variants. **(a)** [ ${}^{13}C$ (2)-FMN]-Fld spectrum, **(b)** [ ${}^{13}C$ (2,4a)-FMN]-Fld spectrum, and **(c)** [ ${}^{13}C$ (2,4a)-FMN]-Fld spectrum with the calculated HYSCORE pattern for a ${}^{13}C$ nucleus, with the parameters specified in the text superimposed in red. Both experimental spectra were taken at the maximum absorption of the EPR absorption, i.e., B=1226.5 mT, τ=112 ns, and T=50 K. Anti-diagonal lines cross the diagonal at the Larmor frequencies $\nu_{\text{14N}}$ , $2\cdot \nu_{\text{14N}}$ , and $\nu_{\text{13C}}$ . The red dotted line in panel **(b)** shows the feature assigned to ${}^{13}C$ (4a) interaction. The two arrows in panel **(c)** point at the two ridges that show the rhombicity of the ${}^{13}C$ (4a) hyperfine tensor. The inset in panel **(b)** shows the orientation selection of the experimental spectra in a sphere octave according to the colors of the accompanying scale. The pulse excitation bandwidth corresponds to 42 MHz.

Q-band HYSCORE experiments were also carried out on [ ${}^{13}C$ (2)-FMN]-Fld and [ ${}^{13}C$ (2,4a)-FMN]-Fld samples, both at the center (Figs. 4 and S1 in the Supplement) and at the tail of the EPR line (Fig. S2 in the Supplement). Again, the larger Larmor frequency of ${}^{13}C$ at the Q-band allows some weak features from echo modulation with ${}^{13}C$ (4a) nuclear frequencies to be seen in the spectra.

The HYSCORE spectra of both samples show intense ridges in the negative quadrant due to ${}^{14}N$ that will be discussed later. Since the focus here is on the ${}^{13}C$ signals, Fig. 4 only shows the positive quadrant of the 2D measurement, set at the center of the EPR line. The spectrum of [ ${}^{13}C$ (2)-FMN]-Fld, on the left, shows a small elongated ridge on the anti-diagonal that crosses the diagonal at the ${}^{13}C$ Larmor frequency. This line is attributed to the hyperfine coupling of ${}^{13}C$ (2), which was estimated to be smaller than 2 MHz. The low-frequency correlations are assigned to ${}^{14}N$ . The spectrum of [ ${}^{13}C$ (2,4a)-FMN] Fld is shown in the center, and next to the described lines, a weak and broad bow-shaped symmetric feature is distinguished on the diagonal, with the knot at about 17 MHz. The feature is surrounded by a dotted line in Fig. 4b. We interpret this feature, undoubtedly associated with ${}^{13}C$ (4a), as the crossing part of two long ridges starting (although not visible) at (40,11) MHz , with the point corresponding to z and the orientation perpendicular to the isoalloxazine plane. The rhombicity of the hyperfine tensor within the plane is manifested by the width of the feature (red area in Fig. 4c), especially in the two ridges reaching the anti-diagonal as indicated by the arrows in Fig. 4c. The points where these structures cross the anti-diagonal allow estimation of the two principal values of the hyperfine tensor in the flavin plane and confirm that the hyperfine couplings in the plane and the hyperfine coupling perpendicular to the plane have opposite signs.

Using the value for $A_{z}$ estimated from the analysis of the ELDOR-detected NMR experiments, the following values are obtained from the analysis and simulation of the spectra for the three principal values of the hyperfine tensor:

$$\begin{array}{c} A_{z}[{}^{13}C(\text{4a})]=(+40\pm 2)\;\mathrm{MHz},\\ A_{x}[{}^{13}C(\text{4a})]=(-13.5\pm 1)\;\mathrm{MHz},\\ A_{y}[{}^{13}C(\text{4a})]=(-9\pm 1)\;\mathrm{MHz}. \end{array}$$

The solid red lines superimposed onto the spectra in the right spectrum are the HYSCORE patterns calculated with the couplings given above. Proper simulations of the spectrum performed with *saffron* are shown in Fig. S1.

### 3.2 Flavodoxin isotopically labeled with ${}^{15}N$ at the FMN ring

#### HYSCORE

Although the Q-band HYSCORE spectra of samples with natural abundance of nitrogen nuclei present intense signals due to hyperfine interactions with these nuclei, their interpretation is difficult, because the ${}^{14}N$ nucleus has a nuclear spin of I=1 and an appreciable quadrupole contribution, which causes the appearance of multiple correlation features (see Fig. 6). The use of samples labeled with ${}^{15}N$ -FMN greatly simplifies the analysis, since its I=1/2 nucleus presents a single nuclear transition per electron spin manifold and therefore a single pair of correlated features per nucleus. The hyperfine parameters obtained from ${}^{15}N$ are directly convertible into those of the ${}^{14}N$ nucleus in the same position.

**Figure 5:**
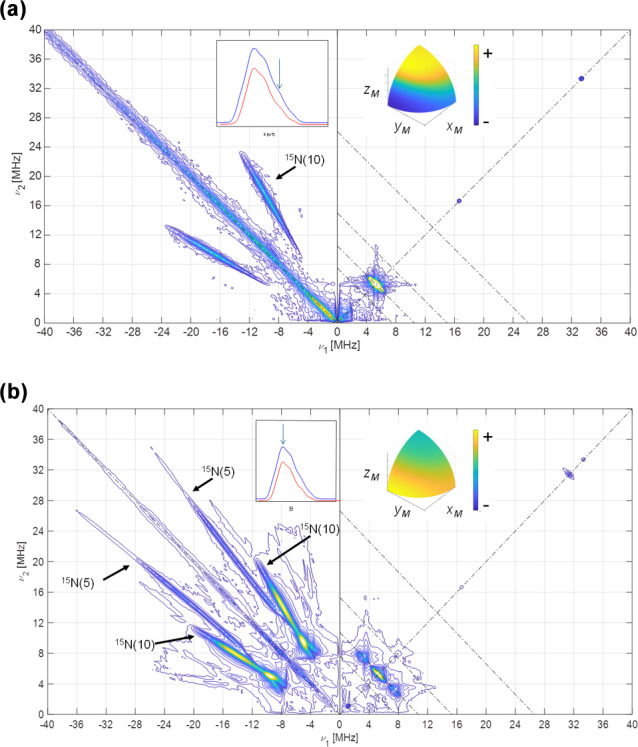
HYSCORE of [ ${}^{15}N$ -FMN]-Fld. **(a)** Spectrum taken at the high-field tail of the EPR absorption spectrum, i.e., B=1219.7 mT. The displayed spectrum is the sum of spectra taken at τ values of 96, 124, 144, and 168 ns. **(b)** Spectrum taken at the absorption maximum of the CW spectrum, i.e., B=1217.2 mT. The displayed spectrum is the sum of spectra taken at τ values of 96, 144, and 168 ns. T=90 K for all of the spectra. The anti-diagonal line crossing the ( + , + ) diagonal at the Larmor frequency $\nu_{\text{15N}}$ has been included for reference. The upper inset on the left shows the echo-detected EPR spectrum of the sample (blue, measured; red, simulated), with the magnetic field setting of the experiment indicated by an arrow. The inset on the right shows the pattern of excited orientations for each spectrum. The pulse excitation bandwidth corresponds to 42 MHz.

The Q-band HYSCORE experiments performed on a [ ${}^{15}N$ -FMN]-Fld sample are displayed in Fig. 5 at the upper tail of the EPR spectrum (Fig. 5a) and at its maximum (Fig. 5b). Only molecules with the magnetic field approximately perpendicular to the flavin plane contribute to the first spectrum (see the inset in Fig. 5a). The hyperfine coupling of N(5) at this orientation is too large (Martínez et al., 1997; Weber et al., 2005) for the excitation bandwidth of the mw pulses to be enough to excite its nuclear frequencies. The spectrum, therefore, only shows a pair of ridges that are symmetrical with respect to the diagonal and approximately parallel to it. These features could be associated with a hyperfine interaction with a nucleus of spin I=1/2 and can attributed to N(10). From the distance between these ridges, the hyperfine interaction of N(10) close to z can be estimated. The short ridge on the ${}^{15}N$ anti-diagonal in the positive quadrant is assigned to the weakly interacting nuclei N(1) and N(3), whose hyperfine couplings have been reported somewhere else (Martínez et al., 2012).

The HYSCORE spectrum at the maximum of the EPR line (Fig. 5b) displays two crossing pairs of correlated ridges. One of them is very long, associated with a highly anisotropic and strong interaction, and assigned to N(5) according to previous results (Martínez et al., 1997; Weber et al., 2005). The other is shorter, overlaps partially with the ridge seen in the parallel spectrum, and is assigned to N(10). Additionally, two pairs of peaks on the ${}^{15}N$ anti-diagonal appear in the positive quadrant at the low-frequency edge of the two ridges, allowing the hyperfine coupling in the plane to be identified as isotropic within the plane. Satisfactory simulations (see Figs. S3 and S4 in the Supplement) were produced using the following axial hyperfine parameters.

$$\begin{array}{c} A_{z}[{}^{15}N(5)]=(+74\pm 3)\;\mathrm{MHz}\\ A_{⊥}[{}^{15}N(5)]=(+5.6\pm 0.3)\;\mathrm{MHz}\\ A_{z}[{}^{15}N(10)]=(+38.0\pm 1.0)\;\mathrm{MHz}\\ A_{⊥}[{}^{15}N(10)]=(+3.2\pm 0.3)\;\mathrm{MHz} \end{array}$$

Here, and in the rest of the article, the errors in the hyperfine parameters were estimated with the aid of multiple calculations of the ridge positions. The optimum parameters were varied from their optimum values, one by one, and the error was set by observing the parameter value that gave a calculated feature whose position was clearly not coincident with the experimental one. The orientation of the tensor cannot be obtained from our results, and the one published by Kay and coworkers was used (Fuchs et al., 2002; Kay et al., 2005) for the simulations.

### 3.3 HYSCORE signals of ${}^{14}N$ on the FMN ring

**Figure 6:**
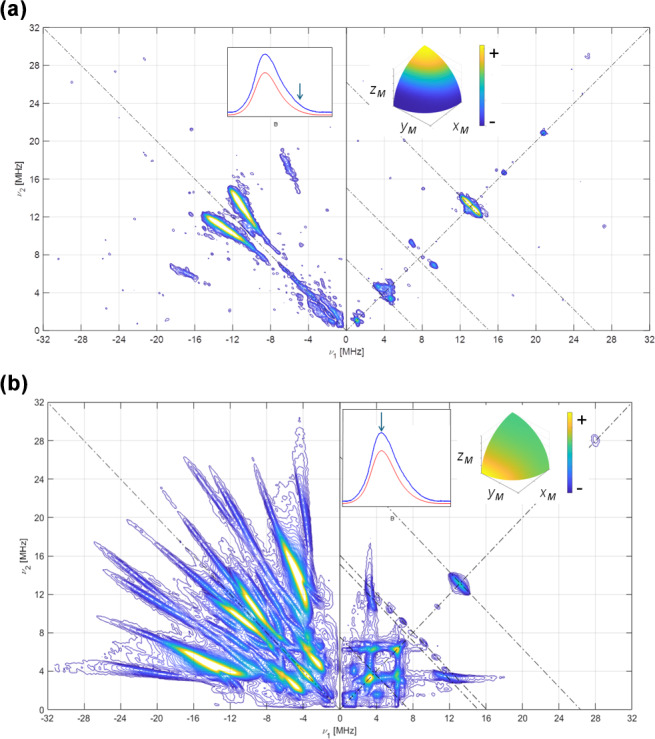
${}^{14}N$ HYSCORE signal Fld samples with naturally abundant nitrogen in the FMN. Spectra were recorded using the sample [ ${}^{13}C$ (2)-FMN]-Fld. **(a)** Spectrum taken at the high-field tail of the EPR absorption, i.e., B=1225.0 mT. The displayed spectrum is the sum of spectra taken at τ values of 96, 112, 128, 144, and 176 ns. **(b)** Spectrum taken at the EPR absorption maximum, i.e., B=1221.0 mT. The displayed spectrum is the sum of spectra taken at τ values of 96, 128, and 208 ns. T=50 K for all spectra. The anti-diagonal lines crossing the ( + , + ) diagonal at the Larmor frequencies $\nu_{\text{14N}}$ , $2\cdot \nu_{\text{14N}}$ , and $\nu_{\text{13C}}$ have been included for reference. The upper inset to the left shows the echo-detected EPR spectrum of the sample (blue: measured; red: simulated), with the magnetic field setting of the experiment indicated by an arrow. The inset to the right shows the pattern of excited orientations for each spectrum. The pulse excitation bandwidth corresponds to 42 MHz.

Once the hyperfine coupling parameters of ${}^{15}N$ are estimated with high precision, they can be transformed directly into those of ${}^{14}N$ in the same position by just applying a factor $g_{N}({}^{14}N)/g_{N}({}^{15}N)=-0.71$ . With the hyperfine parameters already established, the experimental data on the ${}^{14}N$ -FMN can be used to refine the values of the quadrupole tensor. In Fig. 6, the complete set of spectra for [ ${}^{13}C$ (2)-FMN]-Fld is shown. As mentioned before, the spectra are dominated by ${}^{14}N$ ridges. The spectrum at the high-field tail shows two short pairs of ridges assigned to N(10) for the reason mentioned above. It allows a value of 0.8 MHz to be obtained for $\left|Q_{z}\right|$ , i.e., the principal value of the quadrupole tensor in the direction perpendicular to the isoalloxazine ring. The spectrum recorded at the maximum of the EPR absorption contains ridges due to N(10) and N(5) (Fig. 6b). The best simulation of the spectra, shown in Fig. S6 in the Supplement, was produced with the following quadrupole parameters.

$$\begin{array}{c} Q_{z}[{}^{14}N(5)]=1.8\pm 0.1\;\mathrm{MHz}\\ Q_{⊥,1}[{}^{14}N(5)]=-0.8\pm 0.1\;\mathrm{MHz}\\ Q_{⊥,2}[{}^{14}N(5)]=-1.0\pm 0.1\;\mathrm{MHz}\\ Q_{z}[{}^{14}N(10)]=-0.8\pm 0.1\;\mathrm{MHz}\\ Q_{⊥,1}[{}^{14}N(10)]=2.4\pm 0.1\;\mathrm{MHz}\\ Q_{⊥,2}[{}^{14}N(10)]=-1.6\pm 0.1\;\mathrm{MHz} \end{array}$$

Note that the errors in the quadrupole parameters have to be correlated so that the sum of all three principal values is zero. With our data, these principal values cannot be associated with a particular axis in the plane, and we have, therefore, labeled the principal values in the flavin plane as $Q_{⊥,1\text{ or }2}$ .

## 4 Discussion

In this work, the use of hyperfine spectroscopy techniques at the Q-band, in combination with selective isotopic labeling, has proven very useful for experimentally determining the hyperfine couplings of the unpaired electrons of flavoproteins in the semiquinone state, with the nuclei of the isoalloxazine ring having the highest spin densities. The complete set of principal hyperfine values of ${}^{13}C$ (4a), ${}^{15}N$ (5), and ${}^{15}N$ (10) nuclei has been determined. Additionally, the spectroscopic effects of the nuclear quadrupole interaction of ${}^{14}N$ (5) and ${}^{14}N$ (10) have been analyzed. Despite the relevance of these positions in the ring, until now only partial experimental evidence of hyperfine interactions has been available. The Q-band results presented here show that both hyperfine and quadrupolar interactions are compatible with and allow refinement of those previously reported for N(10) and determined using X-band HYSCORE experiments (Martínez et al., 1997). Concerning N(5), the hyperfine structure was never obtained for Fld, but the hyperfine matrix was determined by an analysis of the X-band and W-band CW-EPR spectra, assisted by simulation in the flavoprotein ${\mathrm{Na}}^{+}$ -NQR (Barquera et al., 2003). Here we have shown that direct evidence for the hyperfine interaction with N(5) can also be obtained using Q-band HYSCORE experiments that produce very similar results in Fld. Estimations for the quadrupolar interaction for this nucleus were also obtained for the first time due to the high resolution of the 2D HYSCORE experiments. In spite of the symmetric positions of N(5) and N(10) in the pyrazine central ring, the nuclear quadrupole values differ considerably, possibly indicating a significant contribution of the unpaired electron density, which is considerably larger in the former atom.

Regarding the hyperfine interaction with ${}^{13}C$ , our X-band CW-EPR and Q-band ELDOR-detected NMR and HYSCORE experiments allowed us to determine the complete set of principal values for the hyperfine interaction. Previously, only the two smallest principal values were reported for DNA photolyase (Schleicher et al., 2021); these are in the same range of the ones found here. For the hyperfine couplings of ${}^{13}C$ (2), we also find compatible values for Fld that are always smaller than 2 MHz.

**Table 1:** Comparison between measured and calculated hyperfine parameters for the flavin ring sites with the largest hyperfine couplings.

	Measured isotropic	Calculated isotropic	Relative inaccuracy	Measured anisotropic	Calculated anisotropic
	hyperfine parameter	hyperfine parameter	in a (exp-calc, %)	hyperfine parameters	hyperfine parameters
	a (MHz)^a^	a (MHz)^a^		$T_{x}$ , $T_{y}$ , and ${T_{z}}^{b}$ (MHz)	$T_{1}$ , $T_{2}$ , and ${T_{3}}^{b}$ (MHz)
${}^{13}C$ (4)^c^	-9.7	-11.2	-16 %	-4.1 , -1.5 , +5.7	-0.4 , -1.3 , +1.8
${}^{13}C$ (4a)	$+5.8^{d}$	$+13.2^{c}$	126 %	-14.8 , -19.4 , $+34.2^{d}$	-23.3 , -22.7 , $+46.0^{c}$
${}^{14}N$ (5)	$+20.2^{\text{d,e}}$	$+13.6^{f}$	-33 %	-16.2 , -16.2 , $+32.4^{\text{d,e}}$	-14.6 , -14.6 , $+29.2^{f}$
${}^{13}C$ (5a)^c^	-13.2	-12.3	7 %	-1.9 , +0.2 , +1.6	+1.7 , +2.3 , -3.9
${}^{14}N$ (10)	$+10.5^{\text{d,e}}$	$+7.6^{f}$	-28 %	-8.2 , -8.2 , $+16.5^{e}$	-7.6 , -7.6 , $+15.2^{f}$
${}^{13}C$ (10a)^c^	-14.0	-13.6	3 %	-1.4 , +0.4 , +0.9	+1.1 , -0.7 , -0.5

^a^ Isotropic hyperfine parameter $a=\frac{\left(A_{x}+A_{y}+A_{z}\right)}{3}$ . ^b^ $T_{i}=A_{i}-a$ , i=x,y,z . ^c^ Reference Schleicher et al. (2021). ^d^ This work, in Fld. ^e^ The hyperfine parameters for the ${}^{14}N$ nucleus have been scaled from the ones of ${}^{15}N$ (see the text). ^f^ Reference García et al. (2002).

Our findings can be used for a wide comparison of measured and calculated hyperfine parameters for the main isoalloxazine ring positions in flavoproteins. Table 1 displays a direct comparison between experiments and calculations for nuclei on the ring, bearing the highest hyperfine couplings (4, 4a, 5, 5a, 10, and 10a) where both the isotropic and anisotropic parts of the hyperfine principal values are considered. As indicated in the introduction, measured hyperfine values for different flavoproteins exhibiting neutral semiquinone show just small differences comparable to the variation in the reported calculations (Weber et al., 2001; García et al., 2002; Schleicher et al., 2021).

In the table, it can be seen that the hyperfine interaction of ${}^{13}C$ (4a) predicted by the calculations is significantly overestimated, regardless of whether its isotropic or anisotropic parts are compared. This overestimation represents the most significant discrepancy within the flavin isoalloxazine ring in percentage terms. On the other hand, this could be related to the previously mentioned underestimation of the hyperfine splitting in the ${}^{14}N$ (5) and ${}^{14}N$ (10) nuclei (Weber et al., 2001; García et al., 2002). The isotropic hyperfine constants obtained in the calculations are clearly underestimated for ${}^{14}N$ (5) and ${}^{14}N$ (10) and severely overestimated for ${}^{13}C$ (4a), in which a value more than double that obtained experimentally is predicted. Furthermore, a similar trend occurs when comparing the calculated and measured data for the anisotropic part of the interactions. The calculations reproduce the almost axial character of the hyperfine matrices for the three nuclei well but underestimate their magnitude for ${}^{14}N$ (5) and ${}^{14}N$ (10) (between 10 % and 8 %) and overestimate it for ${}^{13}C$ (4a) (around 35 %).

The values of the isotropic hyperfine parameter calculated for all ${}^{13}C$ nuclei, except for position 4a, reproduce the real values quite well. The overestimation of the isotropic hyperfine coupling of ${}^{13}C$ (4a) in the calculations is quite significant. While the magnitudes of the calculated isotropic couplings of ${}^{13}C$ (4), ${}^{13}C$ (5a), and ${}^{13}C$ (10a) are comparable to that of ${}^{13}C$ (4a), the experimental hyperfine values reveal that the coupling with ${}^{13}C$ (4a) is nearly half that of the others. Regarding the anisotropic part, that of ${}^{13}C$ (4a) remains the largest among the carbon nuclei, but its experimental value makes it comparable to that of the ${}^{14}N$ (5) and ${}^{14}N$ (10) nuclei, despite the fact that the nuclear gyromagnetic factor in these nuclei is quite a bit smaller than that of ${}^{13}C$ .

All this shows that the current calculations present an essential difficulty in realistically describing the electronic spin distribution of the SOMO, which in turn could indicate the need to also improve other aspects of electronic structure prediction. Our findings suggest that the spin density predicted in position 4a could actually be shifted towards the central positions of pyrazine (5 and 10), which may have important consequences for the understanding of the electron transfer mechanisms that specifically involve these positions (Ghisla and Massey, 1989; Lans et al., 2012; Kaya et al., 2025). The fact that the only significant differences between the hyperfine calculations and the experimental values relate to a shift in the electron density between two of the most reactive positions of the flavin cofactor is intriguing, and it remains to be investigated whether this is a general feature of the semiquinone state of flavoproteins or whether it can be hypothetically associated with a modulation of the reactivity by the protein environment. For example, more electron density in position C(4a) would favor reoxidation, whereas more electron density in N(5) would promote hydride transfer (Schleicher and Weber, 2012; Edwards, 2014; Beaupre and Moran, 2020; Guerriere et al., 2025). Whether this modulation is due to a specific protein–flavin interaction or a structural distortion of the isoalloxazine ring is certainly interesting and remains to be identified in future studies. In addition, accurate values of the largest hyperfine splittings in the isoalloxazine ring are critical for characterizing the magnetochemistry involved in the magnetoreception of avian cryptochromes (Hore and Mouritsen, 2016), so the reported experimental values should be very useful for modeling this mechanism.

## Supplement

10.5194/mr-6-183-2025-supplement The supplement related to this article is available online at https://doi.org/10.5194/mr-6-183-2025-supplement.

## Supplement

10.5194/mr-6-183-2025-supplement
10.5194/mr-6-183-2025-supplement The supplement related to this article is available online at https://doi.org/10.5194/mr-6-183-2025-supplement.

## Data Availability

The raw data and MATLAB EasySpin simulation codes are available at 10.20350/digitalCSIC/17281 (Martínez et al., 2025).

## Appendix

The supplement related to this article is available online at https://doi.org/10.5194/mr-6-183-2025-supplement.
