# Supplementary material for: Determining large hyperfine interactions of a model flavoprotein in the semiquinone state using pulse EPR (electron paramagnetic resonance) techniques
Source: Magn Reson (Gott). 2025 Jul 17;6(2):183–97. doi: 10.5194/mr-6-183-2025 (PMC12326372; doi:10.5194/mr-6-183-2025)
Supplement: The supplement related to this article is available online at https://doi.org/10.5194/mr-6-183-2025-supplement. [file mr-6-183-2025-supplement.pdf]

Supplement of Magn. Reson., 6, 183–197, 2025
https://doi.org/10.5194/mr-6-183-2025-supplement
© Author(s) 2025. CC BY 4.0 License.

Open Access
MAGNETIC RESONANCE

Supplement of

Determining large hyperfine interactions of a model flavoprotein in the semiquinone state using pulse EPR (electron paramagnetic resonance) techniques

Jesús I. Martínez et al.

Correspondence to: Inés García-Rubio (inesgr@unizar.es)

The copyright of individual parts of the supplement might differ from the article licence.

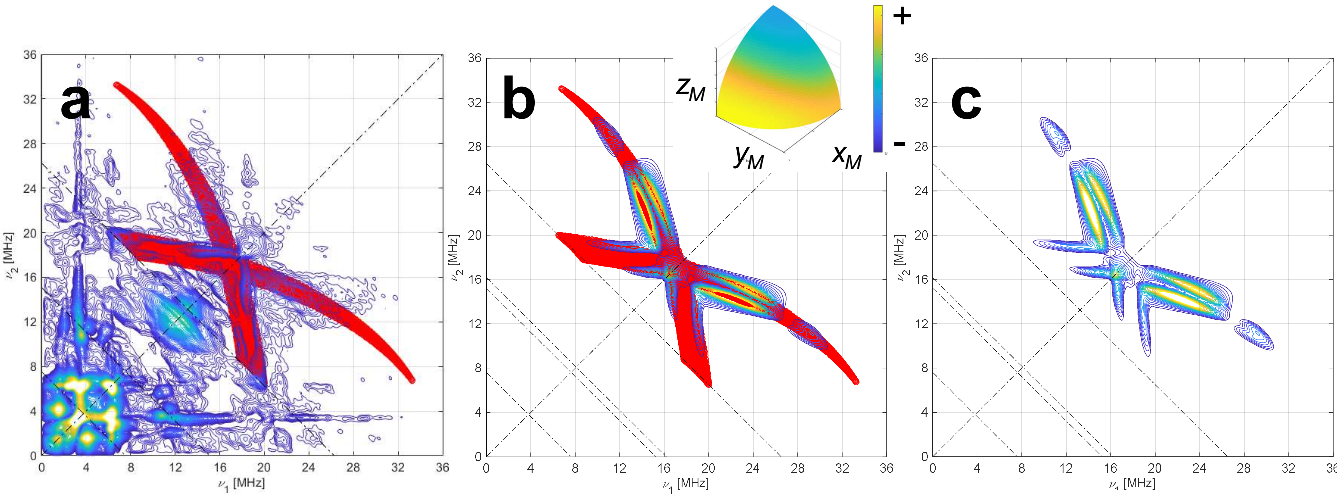

Figure S1: HYSCORE of ^{13}C labelled Fld variants. a) [^{13}C(2,4a)-FMN]-Fld spectrum at the maximum of the EPR absorption (B = 1226.5 mT) with the calculated HYSCORE pattern for a ^{13}C nucleus with the parameters specified in the text superimposed in red, b) Simulated spectrum of ^{13}C(4a) using the function saffron from EasySpin. Superimposed in red, the calculated HYSCORE pattern. c) Simulated spectrum of ^{13}C(4a) using the function saffron from EasySpin. The inset in the central spectrum shows the orientation selection of the experimental spectra in a sphere octave according to the colors of the accompanying scale.

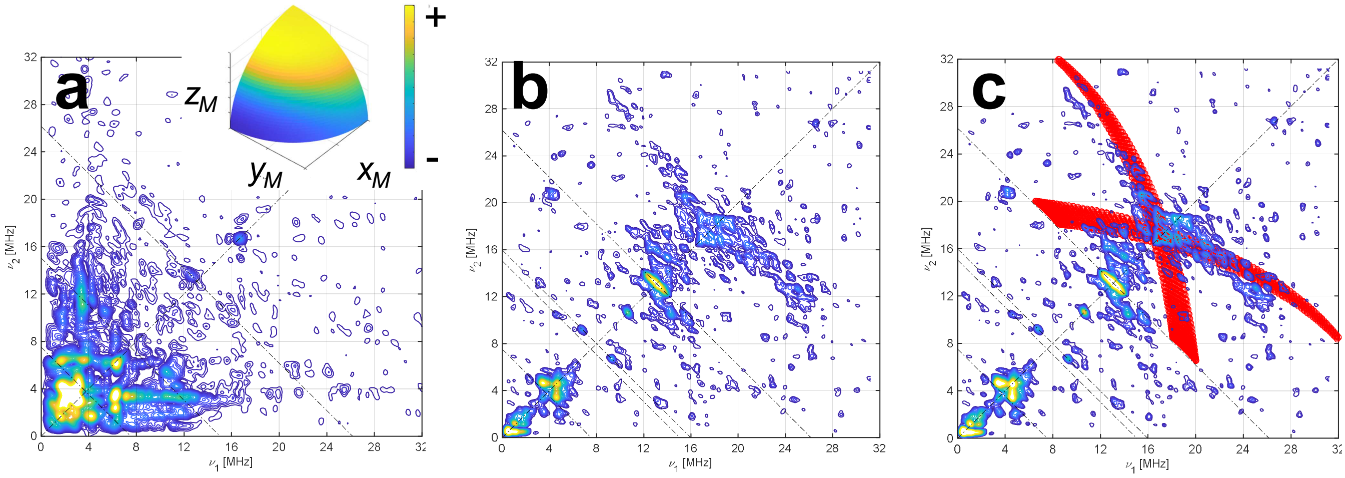

Figure S2: HYSCORE of ^{13}C labelled Fld variants at the high-field tail of the EPR absorption. a) [^{13}C(2)-FMN]-Fld spectrum, b) [^{13}C(2,4a)-FMN]-Fld spectrum, c) [^{13}C(2,4a)-FMN]-Fld spectrum with the calculated HYSCORE pattern for a ^{13}C nucleus with the parameters specified in the text superimposed in red. Both experimental spectra were taken at the high-field tail of the CW-EPR spectrum corresponding to the loose paralel orientation selection shown in the inset of spectrum a. B = 1222.2 mT, τ = 124 ns and T = 50 K for both experimental spectra. Microwave frequency 34.0672 GHz. Antidiagonal lines cross the diagonal at the Larmor frequencies ν_{14N}, $2\cdot\nu_{14N}$ and ν_{13C}.

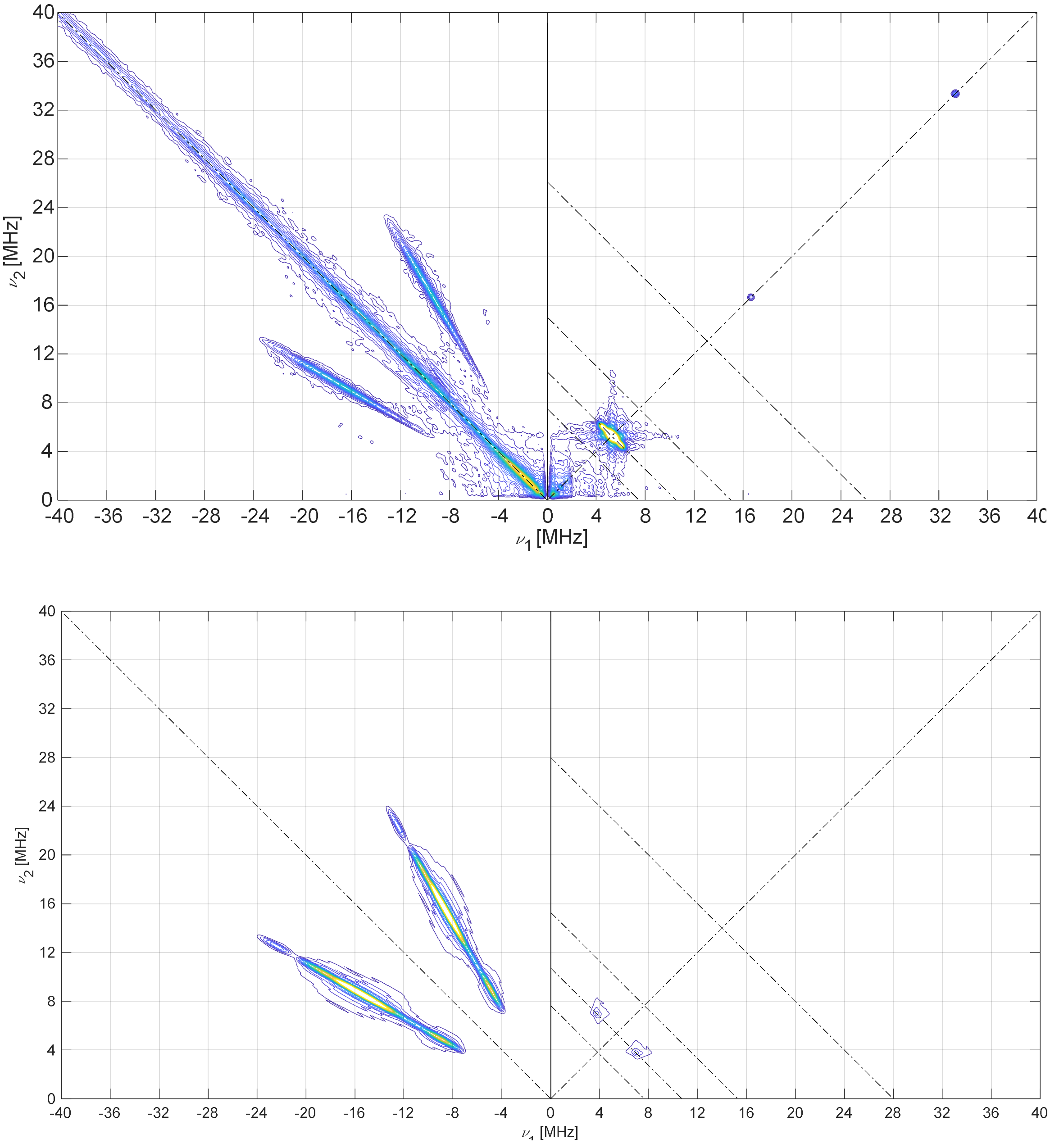

Figure S3: HYSCORE simulation of [^{15}N-FMN]-Fld variant at the high-field edge of the EPR spectrum. a) Experimental spectrum, B = 1219.7 mT, sum of τ values of 96, 124, 144 and 168 ns. It is the same shown in Fig. 5.a of the main text but is depicted also here for comparative purposes. b) Simulation for the same experimental conditions using the parameters specified in the text and in Table 1. The antidiagonal line at the Larmor frequency ν_{15N} has been included for reference.

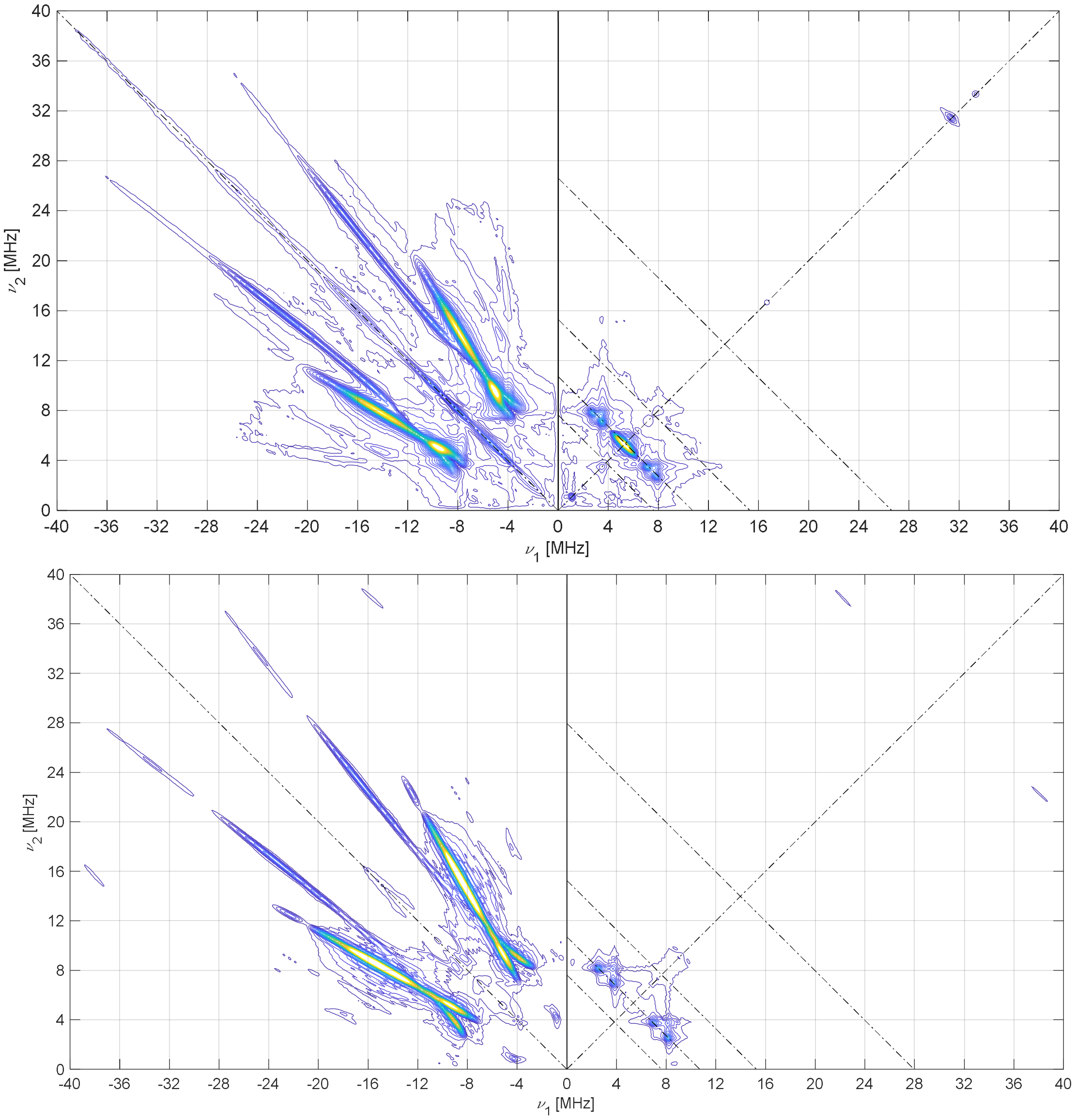

Figure S4: HYSCORE simulation of the [^{15}N-FMN]-Fld variant at the absorption maximum of the EPR spectrum. a) Experimental spectrum, B = 1217.2 mT, sum of τ values of 96, 144 and 168 ns. It is the same shown in Fig. 5.b of the main text but is depicted also here for comparative purposes. b) Simulation for the same experimental conditions using the parameters specified in the text and in Table 1. The antidiagonal line at the Larmor frequency ν_{15N} has been included for reference.

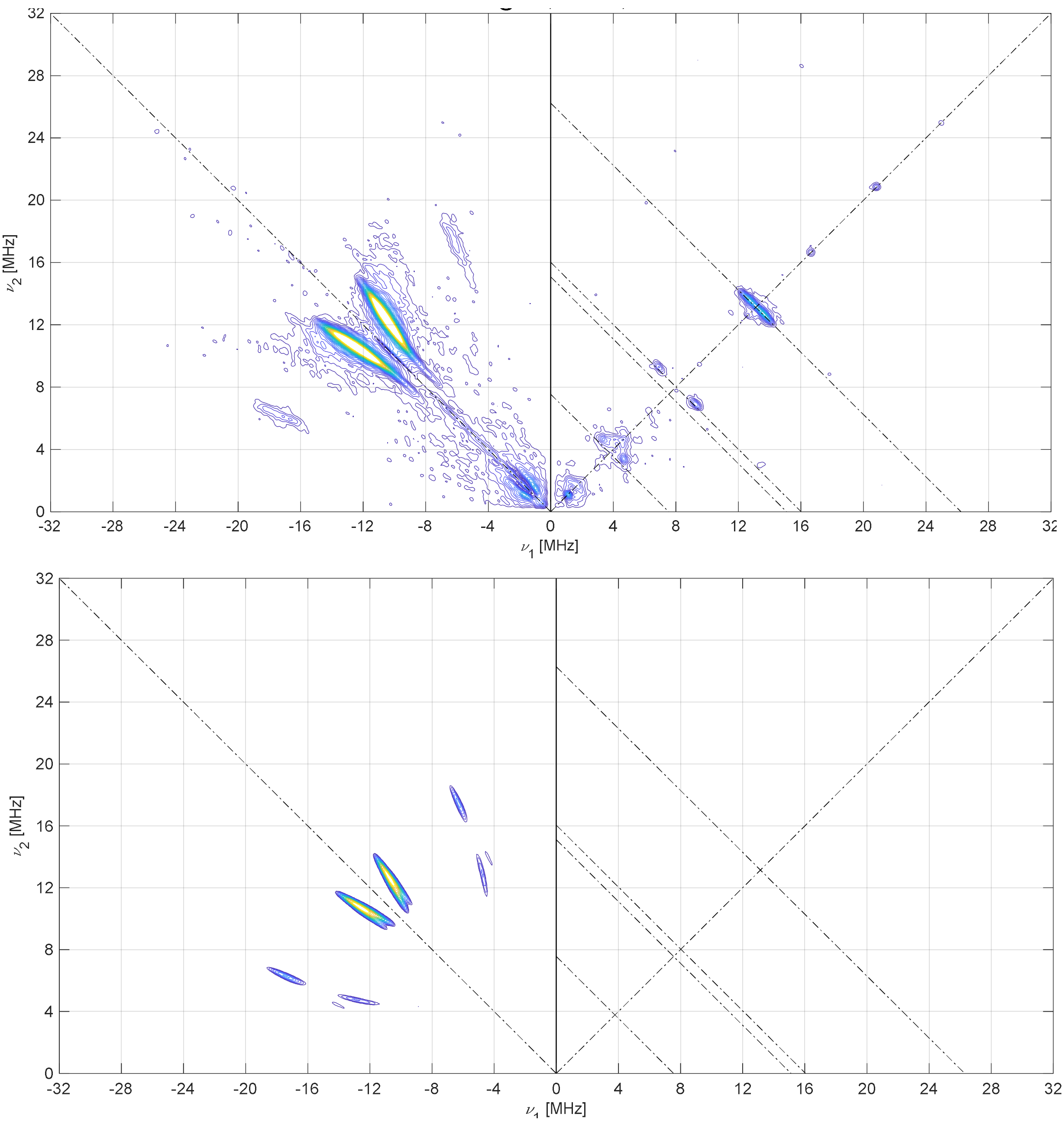

Figure S5: HYSCORE simulation of ^{14}N in [^{13}C(2)-FMN]-Fld at the high-field edge of the EPR spectrum. a) Experimental spectrum, B = 1225.0 mT, sum of τ values of 96, 112, 128, 144 and 176 ns. It is the same shown in Fig. 6.a of the main text but is depicted also here for comparative purposes. b) Simulation for the same experimental conditions using the parameters specified in the text and in Table 1. The antidiagonal line at the Larmor frequencies $\nu_{14N,}$ $2\cdot\nu_{14N}$, ν_{2H} and ν_{13C} have been included for reference.

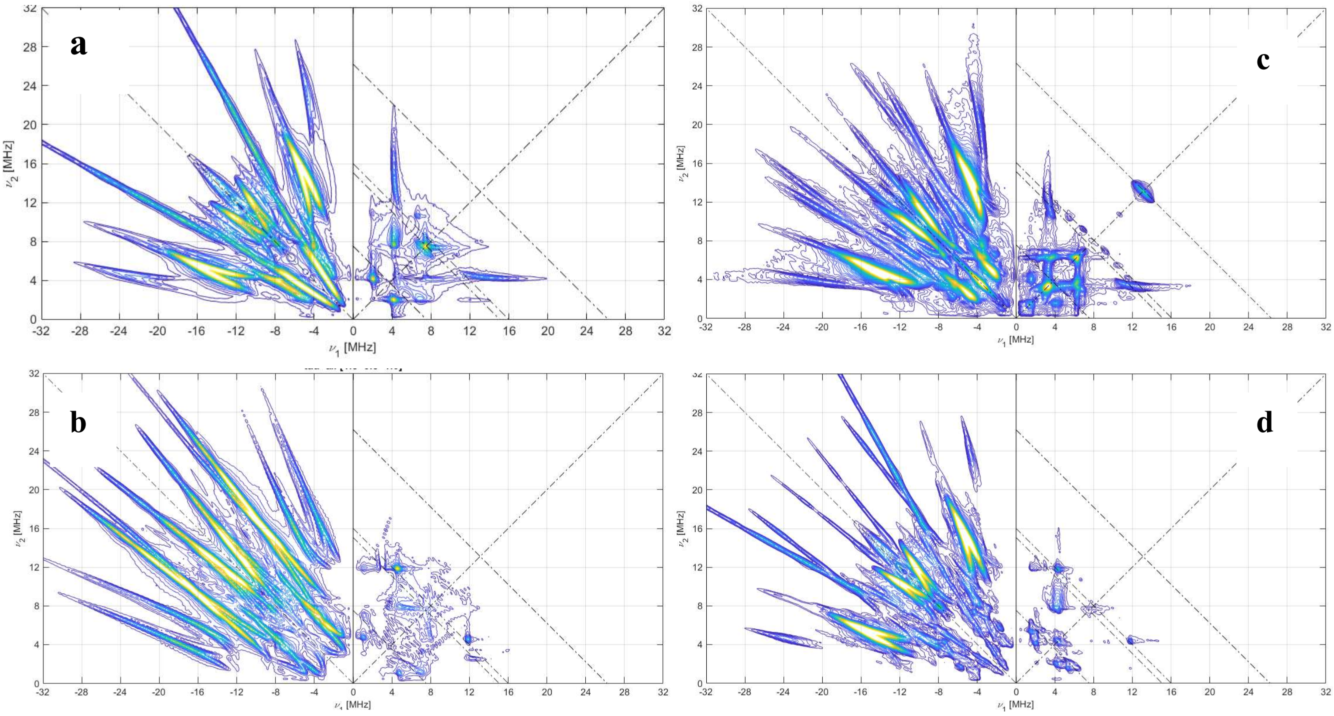

Figure S6: HYSCORE simulations of ^{14}N in [^{13}C(2)-FMN]-Fld at the center of the EPR spectrum. a) Simulation of features associated to N(10), b) Simulation of features associated to N(5), c) Simulation of N(10) and N(5), d) Spectrum obtained from the sum of experimental spectra taken at τ values of 96, 128 and 208 ns, B = 1221.0 mT and T = 50 K. The simulations have been performed using the same τ values together with the coupling parameters specified in the text and in Table 1. The antidiagonal lines crossing the (+,+) diagonal at the Larmor frequencies ν_{14N}, $2\cdot\nu_{14N}$, ν_{2H} and ν_{13C} have been included for reference.
